# The isomiR-140-3p-regulated mevalonic acid pathway as a potential target for prevention of triple negative breast cancer

**DOI:** 10.1186/s13058-018-1074-z

**Published:** 2018-12-11

**Authors:** Anjana Bhardwaj, Harpreet Singh, Celestine Marie Trinidad, Constance T. Albarracin, Kelly K. Hunt, Isabelle Bedrosian

**Affiliations:** 1 Department of Breast Surgical Oncology, The University of Texas MD Anderson Cancer, 1515 Holcombe Blvd, Houston, TX 77030 USA; 2 Department of Pathology, The University of Texas MD Anderson Cancer, Houston, TX USA

**Keywords:** iso-miRNA, miR-140-3p-1, Statin, Aspirin, Dual targeting, AMPK activation, Repurposing, TNBC, Prevention, Preneoplastic, Cholesterol biosynthesis, Metabolic vulnerability

## Abstract

**Background:**

Prevention of triple-negative breast cancer (TNBC) is hampered by lack of knowledge about the drivers of tumorigenesis.

**Methods:**

To identify molecular markers and their downstream networks that can potentially be targeted for TNBC prevention, we analyzed small RNA and RNA sequencing of a cell line model that represent early stages of TNBC development. We have identified direct gene targets of isomiRNA-140-3p and by using cell-based and in vivo model systems we have demonstrated the utility of targeting downstream pathways for prevention of TNBC.

**Results:**

These analyses showed that 5’isomiRNA of miR-140-3p (miR-140-3p-1) and its novel direct gene targets, HMG-CoA reductase (HMGCR) and HMG-CoA synthase 1(HMGCS1), key enzymes in the cholesterol biosynthesis pathway, were deregulated in the normal-to-preneoplastic transition. Upregulation in the cholesterol pathway creates metabolic vulnerability that can be targeted. Consistent with this hypothesis, we found direct targeting of miR-140-3p-1 and its downstream pathway by fluvastatin to inhibit growth of these preneoplastic MCF10.AT1 cells. However, although, fluvastatin inhibited the growth of MCF10.AT1-derived xenografts, histological progression remained unchanged. The cholesterol pathway is highly regulated, and HMGCR enzymatic activity inhibition is known to trigger a feedback response leading to restoration of the pathway. Indeed, we found fluvastatin-induced HMGCR transcript levels to be directly correlated with the degree of histological progression of lesions, indicating that the extent of cholesterol pathway suppression directly correlates with abrogation of the tumorigenic process. To block the HMGCR feedback response to statins, we treated resistant preneoplastic cells with an activator of AMP-activated protein kinase (AMPK), a brake in the cholesterol feedback pathway. AMPK activation by aspirin and metformin effectively abrogated the statin-induced aberrant upregulation of HMGCR and sensitized these resistant cells to fluvastatin.

**Conclusions:**

These results suggest the potential use of combined treatment with statin and aspirin for prevention of TNBC.

**Electronic supplementary material:**

The online version of this article (10.1186/s13058-018-1074-z) contains supplementary material, which is available to authorized users.

## Introduction

Although triple negative breast cancer (TNBC) accounts for ~  5% of the 250,000 annual cases of breast cancer, its more aggressive nature, coupled with its lack of targeted therapy, results in a disproportionate rate of mortality in women with this disease, underscoring the critical need for prevention-based approaches relevant for TNBC. Identification of the micro RNA (miRNA) signatures and their effector pathways that drive early preneoplastic changes are an important first step to developing new strategies for targeted prevention.

There are few resources to characterize the early changes in TNBC tumorigenesis that could be informative to developing novel prevention approaches. We therefore turned to a well-characterized model system generated by outgrowth of mammary epithelial cells initially established from a patient with fibrocystic disease [1]. This model system includes the parental normal-like cell line [MCF10A (P)]; MCF10.AT1, which recapitulates atypia; MCF10.DCIS, which is similar to ductal carcinoma in situ and MCF10.Ca1d, an invasive cancer line [2, 3]. By performing next-generation small RNA and RNA sequencing of this model system, we have recently shown that the majority of miRNA alterations (> 50%) and gene alterations (> 80%) occur during preneoplastic normal to atypia MCF10A(P) to MCF10.AT1 transition [4]. These results suggest that molecular determination of cell fate occurs early in the development of breast cancer, which also creates enormous opportunities for identifying molecular markers and their downstream pathways for prevention of breast cancer.

Lately, numerous RNA sequencing studies have consistently reported the presence of variants of canonical miRNAs called isomiRNAs [5–8]. These isomiRNAs are generated by deletion, substitution, insertion, or a 1 nt shift in the 5′/3′ cleavage site of DICER. The biological and functional relevance of isomiRNAs are only just beginning to be understood. IsomiRNAs have been suggested to share expression characteristics with canonical miRNAs and are equally associated with the translational machinery. A couple of studies have suggested that isomiR-140-3p has functional significance, based on its expression levels in breast cancer cell lines and the fact that targeting this isomiRNA inhibited cell proliferation in breast cancer cell lines [8].

Here, we report that miR-140-3p-1, a variant of miR-140-3p that is generated from a 1 nt shift in pre-miRNA processing by DICER [6], was preferentially expressed during the entire spectrum of preneoplastic progression in the MCF10A-derived TNBC model. We also report on the functional significance of this variant in TNBC tumorigenesis, mediated by regulation of the cholesterol biosynthesis/mevalonic acid (MVA) pathway, which creates a metabolic vulnerability that can be targeted for breast cancer prevention. We demonstrate that targeting the MVA pathway with statins alone elicits a feedback loop that abrogates the potential chemopreventive effect of statin in a TNBC model of breast cancer. However, we show that this feedback loop can be inhibited by activating AMP-activated protein kinase (AMPK) using either aspirin or metformin. These results suggest that combined therapy with statin and aspirin may be needed for effective breast cancer prevention.

## Materials and methods

### Cell lines

We used an MCF10A-based model, a well-established model of TNBC progression developed by Dr Fred Miller (Karmanos Cancer Institute), which recapitulates four major steps of breast cancer progression and comprises normal-like mammary epithelium MCF10A(P), atypical hyperplasia (MCF10.AT1), ductal carcinoma in situ (MCF10.DCIS), and invasive carcinoma (MCF10.Ca1d), representing a stepwise progression in TNBC progression. Normal-like MCF10A (P) is a spontaneously immortalized cell like that was developed using mammary tissue from a women with fibrocystic breast disease. Premalignant MCF10.AT1 cells were obtained by transfection of MCF10A(P) cells with constitutively active oncogene H-ras, which form simple ducts in mice xenografts [3] . Two successive passages of a lesion formed by the MCF10.AT1 cells in xenografts gave rise to MCF10.DCIS cells that forms comedo DCIS lesions in mice xenografts and resembles human DCIS lesions [2]. MCF10.CA1d is a highly tumorigenic derivative of MCF10.AT1 cells [3]. We have tested and found these cells lines to be estrogen receptor (ER), progesterone receptor (PR) and human epidermal growth factor receptor 2 (Her2) negative using the same assays applied for ascertainment of biomarkers in patients’ tumors (data not shown). Others have similarly reported on the biomarker profile of this model, which resembles that of TNBC [9–11]. We purchased MCF10A(P) cells from American Type Culture Collection (ATCC). MCF10.DCIS cells were obtained from Wayne State University, and MCF10.AT1 and MCF10.Ca1d from Karmanos Cancer Institute under a materials transfer agreement. All the cell lines used in the study were authenticated by the source agency and were used within the first 10 passages. Cell lines were periodically tested for mycoplasma and confirmed negative throughout the course of the work presented.

### Generation of MCF10.AT1 (MCF10.AT1-R) cells with adaptive resistance to fluvastatin

While some cells are inherently resistant to statins, others are initially sensitive to statins and eventually develop resistance to statins. Here, we recapitulated adaptive resistance by exposing otherwise sensitive MCF10.AT1 cells to 20 μM fluvastatin. After a period of massive cell death, a cell population that was resistant to fluvastatin emerged and was expanded. These cells were then grown in a maintenance dose of fluvastatin (10 μM). We determined the concentration needed to induce a 50% reduction in the viability (MTT50) of these cells and found that 8.5 μM fluvastatin killed 50% of cells. We designated these resistant cells as MCF10.AT1-R cells, which are four times more resistant than regular MCF10.AT1 cells (MTT50 = 2.1 μM).

### Mice xenografts

Ten million exponentially growing MCF10.AT1 cells that were resuspended in 75 μl PBS and mixed with an equal volume of Matrigel were injected into the mammary fat pads of 5-week-old inbred female BALB/c *Nu/Nu* mice (Charles River Laboratories). All the mice were of the same age and randomly divided in two groups. We injected MCF10.AT1 cells into both flanks. One week after the cell injections, fluvastatin treatment (10 mg/kg body weight/day) was started, and continued for 16 weeks. Fluvastatin was mixed in the drinking water of mice (*n* = 30) and changed every other day. Control mice (*n* = 25) received plain water. Water intake was noted on every change of water, and the concentration of fluvastatin was adjusted to maintain a level of 10 mg/kg body weight/day, if needed. The body weight of mice was noted once a week, and no change in the body weight was observed with statin treatment. Lesion size was measured every week. At the end of 16 weeks, mice were euthanized, and the tissues were explanted from the site of injection. Half of the tissue was fixed in formalin and the other half was saved in TRIzol for RNA extraction. These formalin-fixed tissues were subsequently embedded in paraffin and stained with hematoxylin and eosin to determine their histological grading. Histological grading ranged from simple tubules with 1–2 cell layers (grade 0); simple tubules with > 2 cell layers but no architectural complexity (grade 1); complex hyperplasia (grade 2); atypical hyperplasia (grade 3); to ductal carcinoma in situ (grade 4) as described [10].

### Cloning

The reporter constructs of the 3′UTR of HMG-COA reductase (HMGCR) and HMG-COA synthase1 (HMGCS1) containing the wild-type seed sequence of the miR-140-3p-1 binding site along with 200 flanking nucleotides (both upstream and downstream) were generated from PCR-amplified human genomic DNA that was subsequently cloned downstream of the firefly luciferase open reading frame at the *PmeI* and *XbaI* sites in a pmiRGlo vector (Promega Corporation). Mutant versions of the HMGCR and HMGCS1 3′UTR reporter plasmids were generated by site-specific mutagenesis, as described previously [4]. Sequences of all the primers used are provided in Additional file 1: Table S1.

### Transfection

As described previously (17), MCF10.AT1 and MCF10.DCIS cells were transiently transfected using Lipofectamine 2000 (Invitrogen Technologies) following the manufacturer’s instructions. Cells were plated in 6-well/10-cm culture dishes and then transfected with miR-140-3p-1 mimic (Thermo Scientific) or scramble mimic (10 nM) with/without the pmiRGLo vector containing miR-binding sites. After 5-h incubation in Opti-MEM (Thermo Fisher Scientific), the medium was replaced with regular cell culture medium supplemented with 2X horse serum. Cells were lysed or plated for further assays at 48 h after the transfection.

### RNA extraction and quantitative (q)PCR

Total cellular RNA was extracted from cells using an miRNeasy mini kit (Qiagen) that also preserves small RNAs. Complementary DNA (cDNA) was prepared using an iScript cDNA synthesis kit (Bio-Rad) according to the manufacturer’s instructions. qPCR was performed in triplicate on each sample using an SYBR Green-based PCR assay as described previously [12]. The gene encoding ribosomal protein L19 (*RPL*19) was used as a control to ensure equal loading. All primer sequences are provided in Additional file 1: Table S1. Mature miR-140-3p-1 and miR-140-3p-2 were quantified by using TaqMan-based miRNA assays from Thermo Scientific according to the manufacturer’s instructions.

### Luciferase assay

Luciferase activity was measured in cells that were transfected with empty pmiRGlo vector or 3′UTR-containing reporter vectors using the dual-luciferase reporter system (Promega).

### Cell proliferation

Proliferation of MCF10.AT1 and MCF10.DCIS cells treated with miRNA mimic or drugs was measured either by the MTT dye uptake method or Ki67 antibody based-immunofluorescence assay as described elsewhere [13]. The intensity of Ki67 staining, representing the proliferation index of cells, was measured by counting the cells that expressed high levels (> 3 foci) of Ki67 staining (Ki67-positive cells) or low levels (0–2 foci) of Ki67 staining (Ki67-negative cells).

### Clonogenic survival assay

Colony-forming ability of MCF10.AT1 and MCF10.DCIS cells that were transiently transfected with miRNA mimic or scramble mimic was measured by plating 50–100 cells/well in a regular 6-well culture plate. Cells were plated in their regular medium for 12 days. While testing the effect of fluvastatin, aspirin or metformin drugs were added in the growth medium at indicated concentrations a day after plating the cells. The drugs were not replenished again. After 12 days, cells were stained with 0.5% crystal violet for 5 min. Following staining, dishes were washed twice by inverting them in standing water and then air dried for 1 day. Numbers of colonies were manually counted. A cluster of 40 or more cells (> 2 mm) was considered a colony.

### Western blotting

MCF10.DCIS cells were plated at sub confluent density in 60 mm or 100 mm dishes for western blotting experiments. The day after plating the cells, the regular growth medium was removed and cells were treated with the indicated dose of fluvastatin, aspirin and metformin for 48 h in low-glucose growth medium. Total cellular protein was subjected to SDS-PAGE and transferred to Hybond ECL nitrocellulose membranes (Sigma Aldrich), which were probed with AMPK, pAMPK (Thr 172), HMGCR, or loading control vinculin antibodies. Proteins were detected using an Odyssey Classic infrared imaging system (Li-Cor Biosciences) as described previously [14].

### Ingenuity pathway analysis (IPA)

Top canonical pathways and their effector molecules were generated by IPA (QIAGEN Inc., https://www.qiagenbio- informatics.com/products/ingenuity-pathway-analysis).

### Statistics

MTT data were analyzed using the Kruskal-Wallis test followed by Dunn’s post hoc test. Mice xenograft histological grading data were analyzed by the Fisher exact and chi square tests. All other data were analyzed using Student’s unpaired *t* test.

## Results

### miR-140-3p is lost during breast cancer progression

**Fig. 1:**
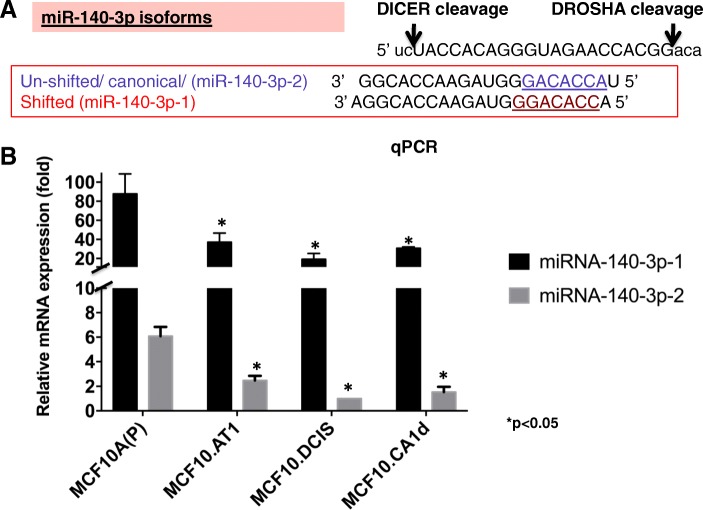
miR-140-3p-1 is lost during breast cancer progression. **a** Sequences of mature miR-140-3p-1 and miR-140-3p-2 isoforms. **b** qPCR showing miR-140-3p-1 and miR-140-3p-2 expression in a MCF10A-based breast cancer progression model. miRNA levels were measured by TaqMan-based qPCR probes. Fold change calculated relative to the cell line with the lowest miRNA expression (highest cycle threshold (Ct)), which was set as 1. Differential miRNA expression for the rest of the comparisons was determined by calculating the fold change of miRNA above this lowest expression level using the Pffafl differential Ct method. Values are also normalized to small nucleolar RNA (RNU44) and represent mean fold change ± SEM

To identify miRNAs that drive normal-to-preneoplastic transition in TNBC progression, we grouped miRNAs according to their expression pattern across the continuum of cell lines in the MCF10A model of TNBC tumorigenesis. Next-generation small-RNA sequencing analyses of this breast cancer progression model, which we have previously published, placed miR-140-3p as one of the top deregulated miRNAs [4]. In order to validate the next-generation sequencing results, we performed qPCR assays using sequence-specific TaqMan-based primers for the canonical miR-140-3p (miR-140-3p-2) and its isomiR, miR-140-3p-1. miR-140-3p-1 is known to be generated by a 1-nucleotide (nt) shift in the cleavage of the miRNA processing enzyme DICER during its processing of pre-miRNA (Fig. 1a). Interestingly, we found miR-140-3p-1 to be expressed at 13-fold to 17-fold higher levels than canonical miR-140-3p-2 throughout the whole spectrum of breast cancer progression, from normal-like MCF10A (P) to preneoplastic MCF10.AT1, DCIS (MCF10.DCIS), and invasive MCF10.Ca1d cells (Fig. 1b). Although the ratio of miR-140-3p-1 relative to miR-140-3p-2 remained consistently higher, the absolute levels of both miR-140-3p-1 and miR-140-3p-2 decreased during TNBC progression, as indicated by qPCR results (Fig. 1b). We found however that the greatest decrease in both miR-140-3p-1 and miR-140-3p-2 occurred early (during the normal (MCF10A.P) -to-atypia (MCF10.AT1) transition) with 60% drop in the levels of both isoforms.

### Restoration of miR-140-3p-1 inhibits cell growth

**Fig. 2:**
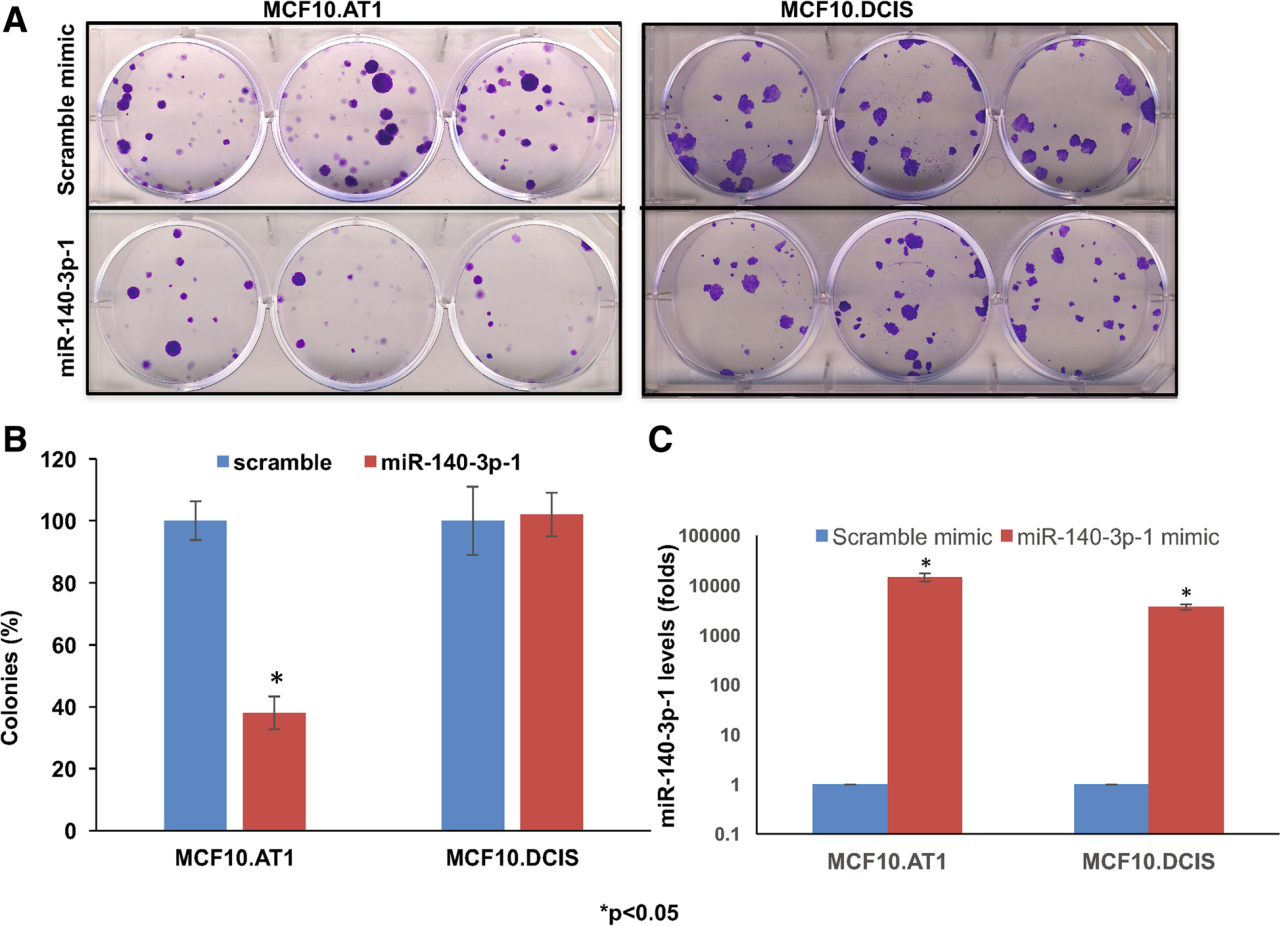
miR-140-3p-1 inhibits the colonization ability of breast preneoplastic cells. **a** Clonogenic survival assay showing reduction in the number of colonies formed by preneoplastic MCF10.AT1 and MCF10.DCIS cells with the transient transfection of a miR-140-3p-1 mimic relative to a scramble control mimic. **b** Quantification of percentage of inhibition in colonizing ability of AT1 and DCIS cells with miR-140-3p-1 transfection normalized to scramble control mimic transfection. **c** Transfection followed by qPCR assay showing the transfection efficiency of MCF10.AT1 and DCIS cells. Both the cell lines express mature miRNA-140-3p-1 more than 3500 times relative to scramble control mimic. Cells were treated with indicated mimics for 48 h and the total cellular RNA obtained was analyzed for expression of mature miR-140-3p-1 by qPCR. Values represent mean fold change ± SEM. *p < 0.05

Although much is known about a myriad of biological functions performed by canonical mature miRNAs, understanding of the relevance of isomiRNAs remains elusive. Therefore, to investigate the role of miR-140-3p-1, we ectopically expressed this isomiRNA in breast preneoplastic (MCF10.AT1) and MCF10.DCIS cells and measured its effects on the colonizing ability of cells, as described in “Materials and methods”. We found ectopic expression of miR-140-3p-1 to preferentially inhibit the colonization ability of preneoplastic MCF10.AT1 cells (62% reduction, *p* < 0.05 compared to control) but did not have functional effect further down the disease progression spectrum in MCF10.DCIS cells (Fig. 2). Similarly, ki-67 staining indicated that miR-140-3p-1 restoration modestly inhibited the cell proliferation (by 9.8%, *p* = 0.08) of preneoplastic MCF10.AT1 cells but not in MCF10.DCIS cells (Additional file 2: Figure S1). In order to confirm that these changes represented biologically relevant differences across the cell lines rather than a consequence of variability in transfection efficiency, we transfected both MCF10.AT1 and MCF10.DCIS cells with miRNA-140-3p-1 or scramble control mimic. qPCR analysis of these cells suggested both the cell lines to be expressing more than 3500-fold expression of miR-140-3p-1 relative to scramble control (Fig. 2c), thus confirming that the growth inhibition seen with miR-140-3p-1 preferentially in MCF10.AT1 represents important context-specific effects of miR-140-3p-1 treatment.

### miR-140-3p-1 directly binds and regulates *HMGCR* and *HMGCS1*

**Fig. 3:**
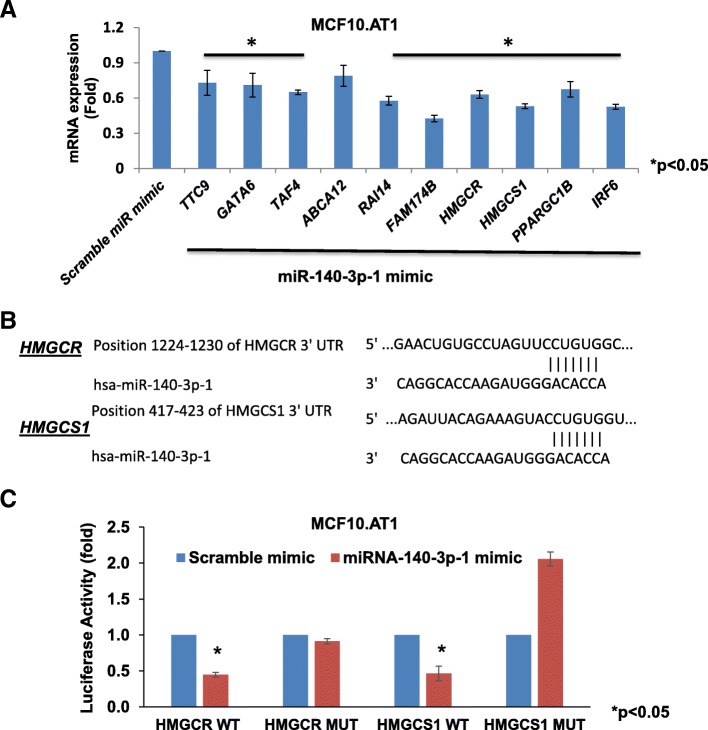
Cholesterol pathway mediators HMG-COA reductase (*HMGCR*) and HMG-COA synthase1 (*HMGCS1*) are directly regulated by miR-140-3p-1. **a** qPCR results showing the effect of transfection of miR-140-3p-1 mimic on its predicted target genes relative to their expression, with scramble control mimic transfection that was set as 1. MCF10.AT1 cells were treated with scramble mimic and miR-140-3p-1 mimic for 48 h and the total cellular RNA was analyzed for expression of indicated mRNAs by qPCR. Values are normalized to RPL19 mRNA levels and change in target gene expression calculated under treatment conditions as fold change greater than the expression levels seen in control (mimic) treatment. **b** Schematic showing TargetScan-predicted binding sites of mature miR-140-3p-1 in the 3’ UTR of *HMGCR* or *HMGCS1*. **c** Luciferase assay showing normalized firefly expression in MCF10.AT1 cells 48 h after being transfected with the wild-type (WT) or mutated (MUT) *HMGCR* or *HMGCS1* reporter along with the miR-140-3p-1/scramble mimic. A Renilla luciferase construct was co-transfected to control for transfection efficiency; the mean values shown are from three independent experiments. Values represent mean fold change ± SEM. *p < 0.05

To identify functional gene targets and driver pathways downstream of miR-140-3p-1, we integrated the expression pattern of miR-140-3p-1 with the gene expression data obtained by the next-generation RNA sequencing that we previously performed [4] on the MCF10A model system. In particular, we focused on the TargetScan predicted gene targets of miR-140-3p-1 that were upregulated significantly during the progression from the non-cancer parental line [MCF10A(P)] to MCF10.DCIS. The filters that we employed to obtain functionally relevant predicted gene targets of miR-140-3p-1 are listed in Additional file 3: Figure S2A. From these analyses, we identified 10 genes that have significant (*p* < 0.05) and reverse correlation with miR-140-3p. Ingenuity pathway analysis showed that the mevalonate/cholesterol biosynthesis pathway, through its key gene mediators *HMGCR* and *HMGCS1*, was the top predicted pathway to be deregulated (Additional file 3: Figure S2 B). As a first step, we validated the endogenous levels of *HMGCR* and *HMGCS1* transcripts in the MCF10A progression model using qPCR. These analyses showed a steady increase in the levels of *HMGCR* (about 2.5-fold) and *HMGCS1* (5.5-fold) in the cell lines from later stages of tumorigenic progression compared to the normal-like MCF10A(P) cell line (Additional file 3: Figure S2 C&D). Next, to test whether *HMGCR* and *HMGCS1* are regulated by miR-140-3p-1, we restored levels of these in the preneoplastic MCF10.AT1 cell line by transfecting cells with miR-140-3p-1 mimic. These qPCR-based assays showed that indeed *HMGCR* and *HMGCS1* were repressed (by 37% and 47%) with the addition of miR-140-3p-1 mimic relative to their expression upon transfection of scramble control mimic, confirming this predicted miR-gene relationship to be valid in the context of breast preneoplastic cells (Fig. 3a). Finally, to test whether miR-140-3p-1 directly binds to and regulates *HMGCR* and *HMGCS1*, we cloned a 500-bp fragment of the *HMGCR* and *HMGCS1* 3’UTR containing the miR-140-3p-1 binding site in pmiR-Glo, a luciferase vector. As expected, we found the miR-140-3p-1 mimic to repress the reporter luciferase activity of the wild-type construct (containing the intact miR-140-3p-1 binding site from the *HMGCR* and *HMGCS1* 3’ UTR) by 55% (Fig. 3c). As a control, we also studied the effect of miR-140-3p-1 mimic on luciferase activity of constructs that harbor a mutated miR-140-3p-1 binding site from the 3’ UTR of *HMGCR* and *HMGCS1*. As expected, the miR-140-3p-1 mimic failed to repress the luciferase activity of the mutant constructs (Fig. 3c), indicating that miR-140-3p-1 directly binds to its binding site in the 3’UTR of *HMGCR* and *HMGCS1* and represses their activity.

### MVA pathway targeting inhibits tumorigenic properties in vitro

**Fig. 4:**
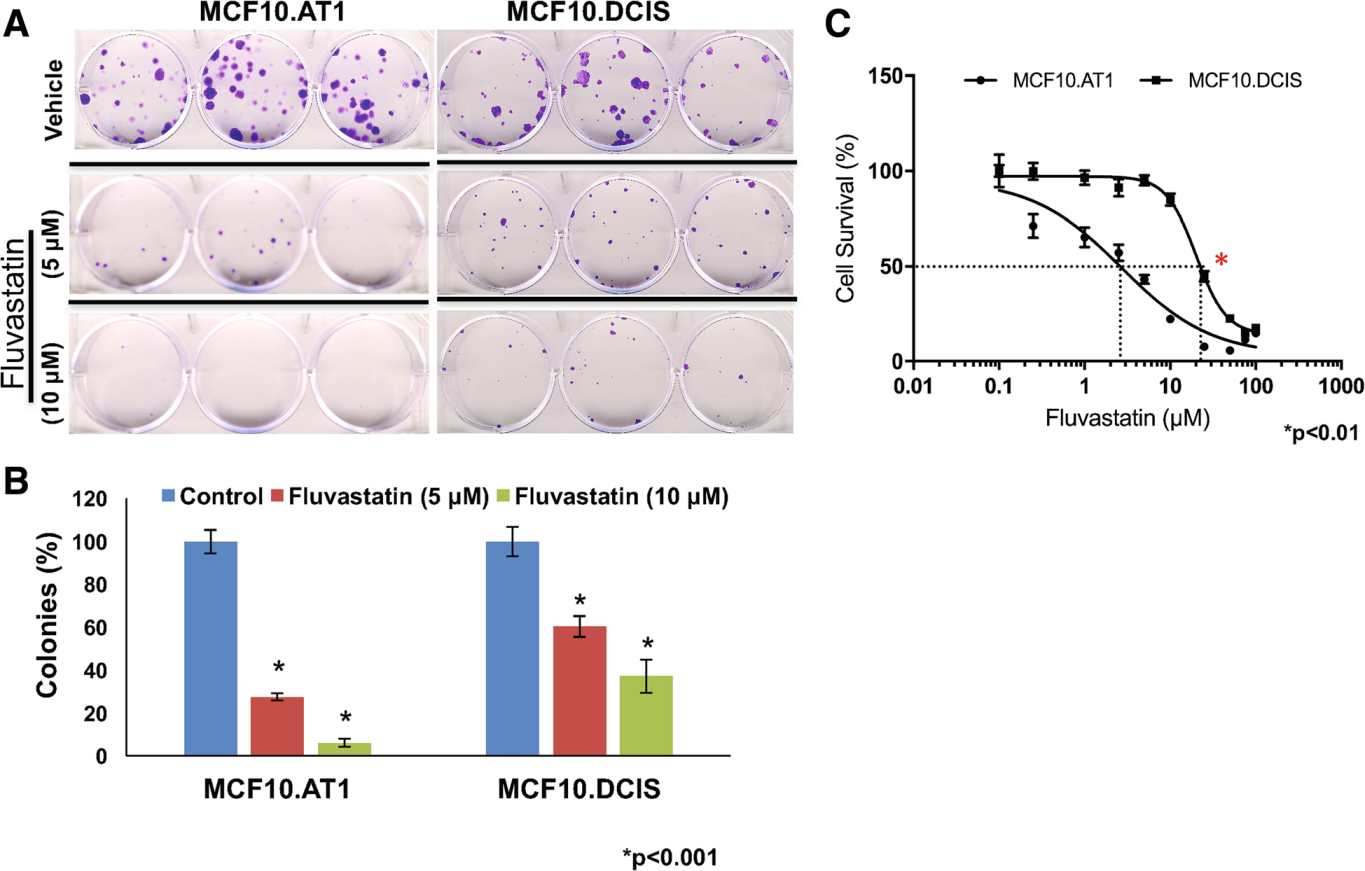
Fluvastatin inhibits colonization ability and proliferation of breast preneoplastic cells. **a** Clonogenic survival assay showing crystal violet-stained colonies formed by preneoplastic MCF10.AT1 and MCF10.DCIS cells after 12 days of treatment with fluvastatin or vehicle control. **b** Quantification of colonies formed by MCF10.AT1 and MCF10.DCIS cells treated with fluvastatin or vehicle control. Values represent number of colonies (%) ± SEM. *p < 0.001. **c,** Dose-response curves of MCF10.AT1 and MCF10.DCIS cells showing significantly different half maximal inhibitory concentration values across these two cells lines (2.1 vs 18, *p < 0.01.), as measured by the MTT dye uptake method after 48 h of vehicle or fluvastatin treatment. Values represent cell survival (%) ± SEM

Because statins inhibit the activity of HMG-CoA reductase, we next investigated whether targeting this pathway with fluvastatin impairs the growth of breast preneoplastic (MCF10.AT1) and DCIS (MCF10.DCIS) cells. Fluvastatin (5 μM and 10 μM) impaired the cell colonizing ability of both cell lines but was more effective in preneoplastic AT1 cells (by 72.42% and 93.9% relative inhibition for 5 μM and 10 μM doses, respectively, compared to vehicle control) than in DCIS cells (by 39.66% and 62.76% relative inhibition for 5 μM and 10 μM doses, respectively, compared to vehicle control) (*p* < 0.001) (Fig. 4a, b). Similarly, an MTT assay also revealed that fluvastatin preferentially inhibited the cell proliferation of MCF10.AT1 cells as indicated by an IC50 of 2.1 μM in MCF10.AT1 cells relative to half maximal inhibitory concentration (IC50) of 18 μM in MCF10.DCIS cells (*p* < 0.01), values that are derived from the fluvastatin dose response curves shown in Fig. 4c.

### MVA pathway targeting in mice xenografts

**Fig. 5:**
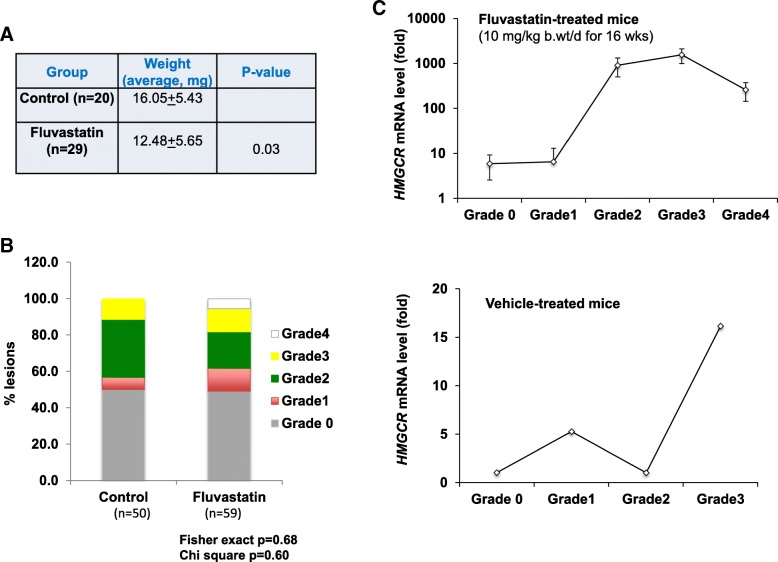
Fluvastatin treatment moderately inhibits growth of AT1-derived lesions. **a** Average weights of the MCF10.AT1 -driven xenografts from mice treated with fluvastatin or vehicle control. **b** Distribution of lesion grades within the xenografts, classified as histological grades 1 through 4, in control (*n* = 50 lesions) and fluvastatin-treated (*n* = 59) groups (*n* = 59 lesions) were similar in both groups (*p* = 0.68). **c** qPCR data showing the relative HMG-COA reductase (*HMGCR*) mRNA levels (normalized to ribosomal protein (*RPL*)19) in the lesions of vehicle-treated or fluvastatin-treated mice sub-grouped by histological grade after 16 weeks of treatment. Grade-0 samples, which had the lowest expression level (highest cycle threshold (Ct)) value were set as 1 and the differential mRNA expression for the rest of the samples was determined by calculating the fold change of mRNA above this lowest value using the Pffafl differential Ct method. Values represent mean fold change ± SEM

To test the efficacy of fluvastatin in inhibiting the progression of MCF10.AT1-driven lesions in mice xenografts, we injected MCF10.AT1 cells in both the left-side and right-side mammary glands of 55 inbred nude (BALB/c *Nu/Nu* homozygous) mice. Thirty mice were given fluvastatin (10 mg/kg body weight/day) in their drinking water. This dose was selected as the human equivalent of this dose (48 mg for an adult weighing 60 kg, using the BSA normalization method [15]) is well within the prescribed clinical dosing for fluvastatin. As a control group, the remaining 25 mice were given plain water. After 16 weeks of treatment, mice were euthanized, and the lesions were collected from the sites of the implant. Although we found fluvastatin-treated lesions to be 25% smaller than vehicle treated lesions, as indicated by their average weight (16.05 vs 12.48 mg, *p* = 0.03) (Fig. 5a), the histological findings were similar between these two groups. Specifically, using the grading system developed by Visscher et al. and as described in the “Materials and methods”, we saw no difference in the distribution of histologic grade between the treated and control xenografts (Fig. 5b), indicating that statin treatment did not inhibit the progression of MCF10.AT1-driven lesions. Given this lack of efficacy, we next investigated whether statins effectively inhibited the target mevalonate/cholesterol pathway. Although assessment of HMGCR inhibition is typically performed using blood assays, earlier studies suggest that in at-risk women, dysregulation of the MVA pathway occurs in the tissue independent of blood levels [16]. Therefore, in order to understand the local tissue effects of fluvastatin treatment, we chose to measure messenger RNA (mRNA) levels of *HMGCR* within the explanted xenografts. Statin inhibition of HMG-CoA reductase enzymatic activity in normal cells and statin-sensitive cancer cells [17] is known to activate a series of feedback responses, including modulation of AMPK, which in turn fine-tunes the levels and activity of *HMGCR* and Sterol regulatory element binding protein (*SREBP*)1 and *SREBP*2, leading to homeostatic levels of the cholesterol pathway [17–19]. Analyses of HMGCR levels in the explanted xenografts, to our surprise, revealed that lesions from the fluvastatin treatment group of our mouse xenografts did not show uniform suppression of *HMGCR* mRNA (Fig. 5c). Rather we found a steady increase in *HMGCR* with increase in histologic grade of progression; the least progressed histological grade lesions (grade 0 and grade 1) expressed lower levels of *HMGCR* transcript, indicating more effective inhibition of the cholesterol pathway. Conversely, we found about 200-fold higher levels of *HMGCR* in higher-grade lesions (grade 2 and grade 3) in the fluvastatin-treated group. In contrast, the basal levels of *HMGCR* transcript was relatively uniform in the vehicle-treated xenografts (maximum 16-fold change across lesions compared to 200-fold in the statin-treated group), suggesting that the variation in *HMGCR* mRNA levels in the fluvastatin group reflects the normal homeostatic cellular response to inhibition of the cholesterol biosynthesis pathway.

### Aspirin sensitizes resistant cells to fluvastatin through AMPK activation

**Fig. 6:**
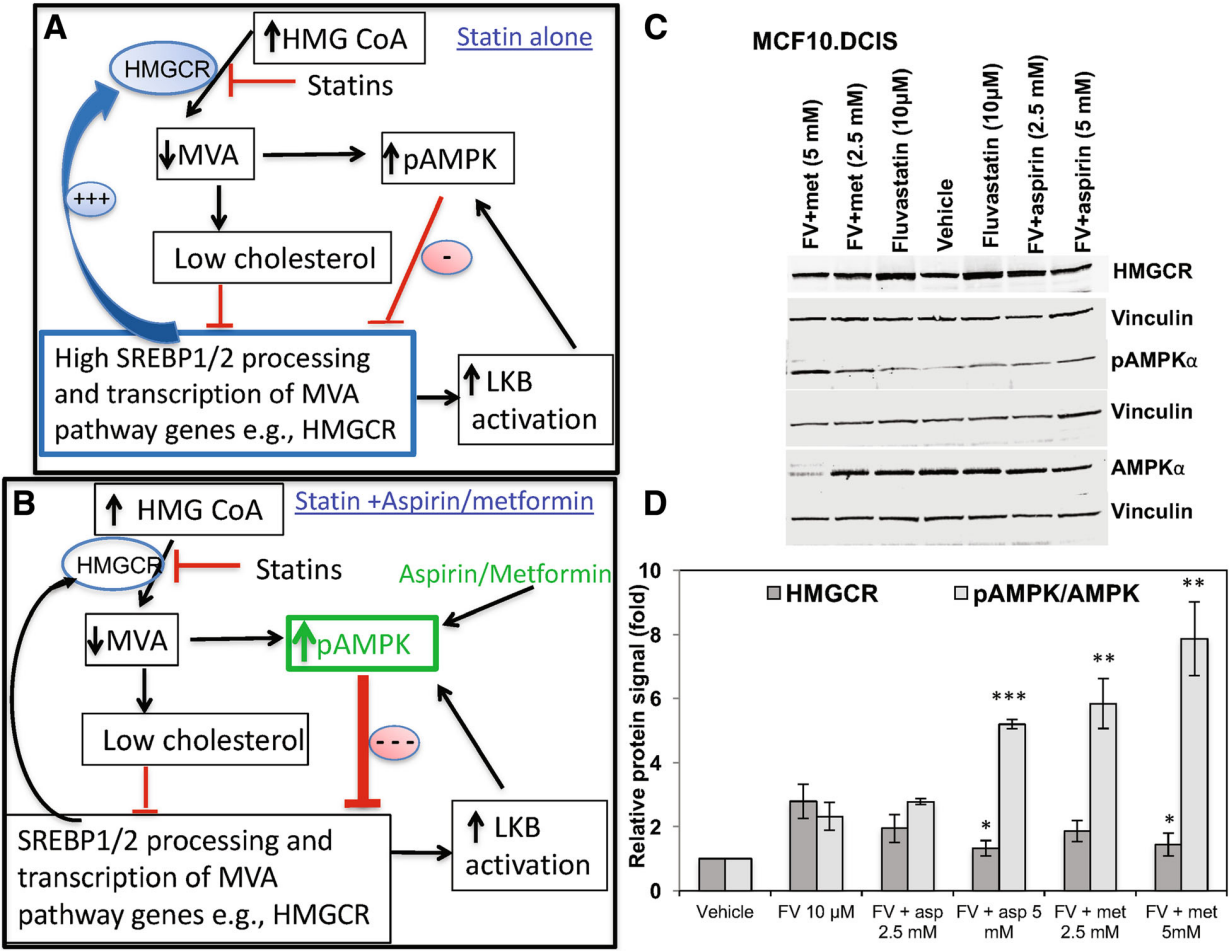
A combination of fluvastatin and aspirin/metformin activates pAMPK and effectively inhibits HMG-COA reductase (HMGCR) in MCF10.DCIS cells. **a**, **b** The mevalonate pathway, where statin treatment triggers a restorative feedback loop leading to upregulation of *HMGCR* mRNA levels (**a**) and aspirin and metformin put a brake in this restorative feedback loop through activation of AMPK phosphorylation (**b**) **c** Western blot showing the endogenous levels of the indicated proteins in the lysates of MCF10.DCIS cells treated with vehicle/fluvastatin (FV) in combination with aspirin or metformin (met) for 48 h. Vinculin was used as the loading control. **d** Averaged densitometry across multiple western blots for the indicated proteins showing the effect of fluvastatin alone or in combination with aspirin (asp) or metformin (met) on HMGCR and pAMPK levels. HMGCR levels were calculated by normalizing to the loading control vinculin. pAMPK levels were first normalized to vinculin and next a ratio of normalized pAPMK was obtained by dividing with normalized levels of total AMPK. Values represent mean fold change ± SEM. *p < 0.05, **p < 0.01 and ***p < 0.001 as compared to fluvastatin alone

If, indeed over activation of the MVA pathway feedback loop and thus insufficient suppression of HMGCR contributed to resistance to statins (Fig. 6a), we predicted, based on the known feedback mechanism that AMPK-activating drugs, such as aspirin and metformin, will inhibit this feedback response and potentiate the ability of statins to inhibit HMGCR (Fig. 6b), leading to more effective abrogation of cell growth. To test this, we treated MCF10.DCIS cells, which are relatively resistant to statins (IC50 of MCF10.DCIS cells was significantly higher than that of MCF10.AT1 cells, *p* < 0.01, Fig. 4c) with a combination of fluvastatin and varying concentrations of the AMPK-activating drugs aspirin and metformin for 48 h. Fluvastatin treatment induced HMGCR protein expression (Fig. 6c and d). As postulated, aspirin and metformin substantially abrogated the fluvastatin-induced HMGCR protein expression by 50% (2.8-fold increase in HMGCR with fluvastatin vs 1.3 to 1.4-fold increase with the combination therapy as compared to vehicle treatment, Fig. 6c and d). In tandem, we found that aspirin and metformin increased levels of pAMPK. This was confirmed by western blot analyses that demonstrated a dose-dependent increase in pAMPK levels with aspirin (2.77-fold to 5.18-fold) and metformin (5.83-fold to 7.8-fold) (Fig. 6c and d). Compound C blocked the aspirin-induced pAMPK activation and consistently led to a partial restoration in HMGCR levels (data not shown). These data are consistent with the model wherein the homeostatic feedback loop, restoring HMGCR levels, can be blocked by activation of pAMPK (Fig. 6b).

**Fig. 7:**
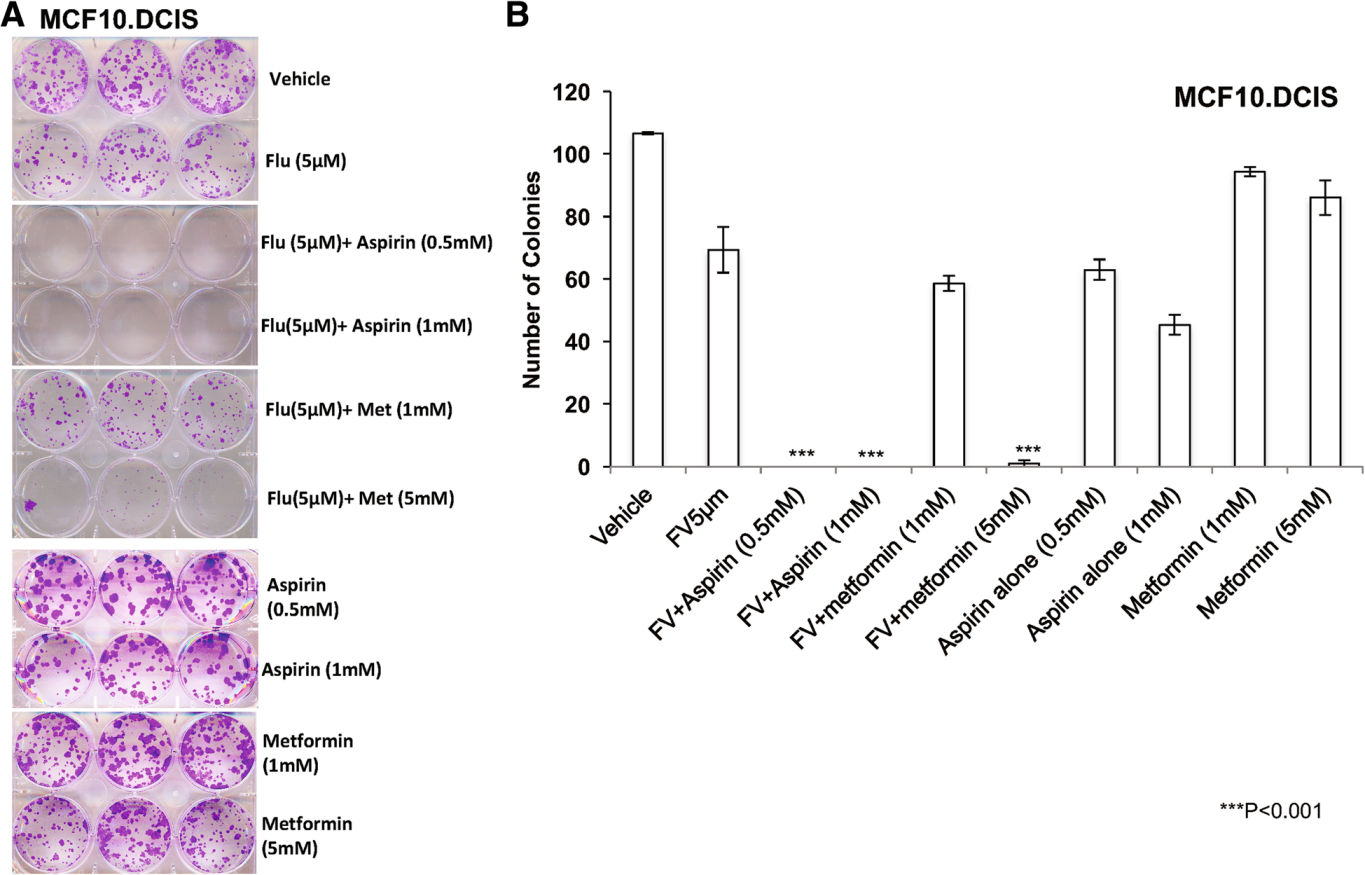
Aspirin and metformin overcome inherent resistance of MCF10.DCIS cells. **a**, **b** Images and quantification of a clonogenic survival assay showing reduction in the number of colonies formed by inherently resistant MCF10.DCIS cells treated with fluvastatin (Flu (**a**)) alone or fluvastatin (FV (**b**)) in combination with aspirin or metformin relative to vehicle control. Colonies ≥ 2 mm were scored and tabulated. Values represent average number of colonies ± SEM. Indicated drug combinations were significantly different from fluvastatin alone; ***p < 0.001

We next tested the functional significance of dual targeting of the cholesterol pathway. We performed colony formation assays, treating DCIS cells with aspirin (0.5 mM and 1 mM) in combination with fluvastatin (5 μM) and found that such dual treatment completely inhibited the ability of cells to form colonies at both 0.5 mM and 1 mM dose of aspirin (100% inhibition, Fig. 7a and b, *p* < 0.001), compared to single-agent treatments. Similarly, combination treatment with fluvastatin (5 μM) and metformin (5 mM) also substantially reduced colony formation more than fluvastatin and metformin alone (Fig 7a and b, *p* < 0.001). Although treatment with fluvastatin alone had modest efficacy (35% inhibition in total colonies), a combination of fluvastatin and aspirin or metformin was most effective (100% inhibition by aspirin and 99% inhibition by metformin (5 mM)) to overcome the inherent resistance to statin seen in DCIS cells. This is consistent with the reported complexities of MVA pathway regulation due to feedback activation loops, which leads to requirement of blockage of need to block this pathway at two nodes in order to completely inhibit this pathway, to inhibit cellular growth [20].

**Fig. 8:**
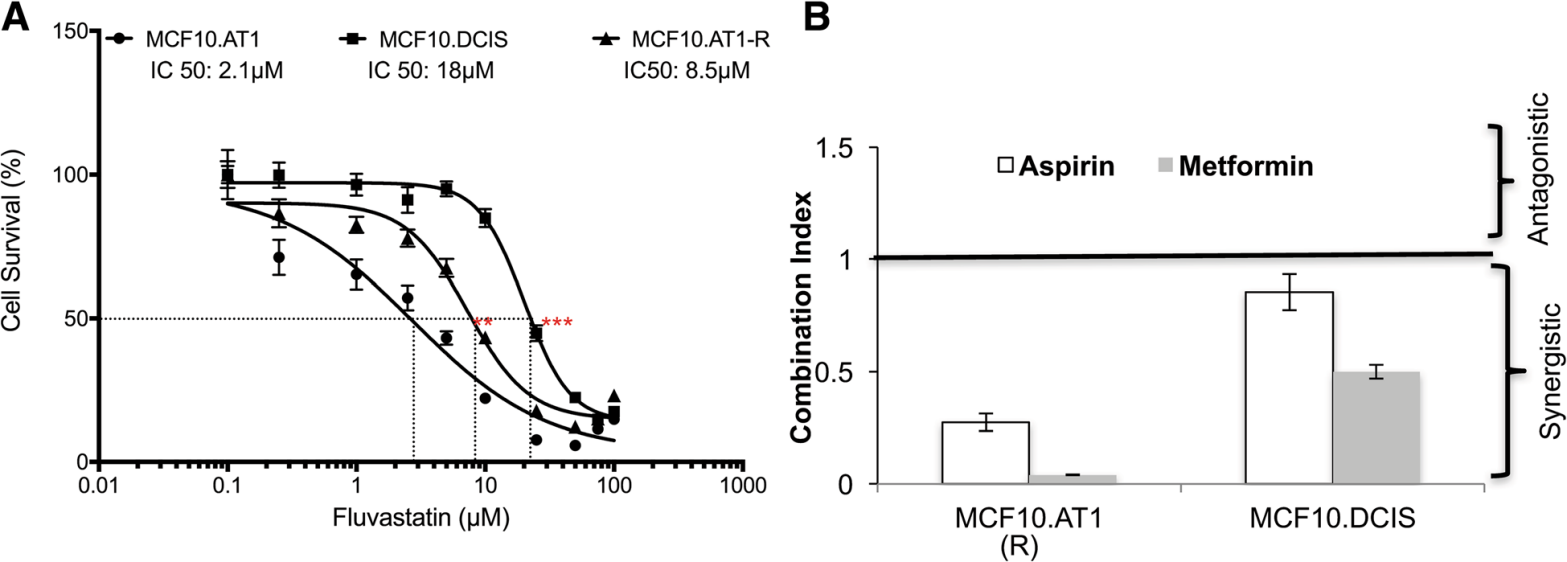
Combination treatment with fluvastatin and aspirin/metformin synergistically inhibits cellular proliferation of MCF10.AT1-R and MCF10.DCIS cells. **a** Dose-response curves showing proliferation of MCF10.AT1-R cells that have developed resistance to fluvastatin (adaptive resistance) in comparison to its parental MCF10.AT1 cells and an inherently resistant MCF10.DCIS cells as measured by the MTT dye uptake method after 48 h of vehicle or fluvastatin treatment. **b** Combination index (CI) as determined using the Calcusyn software, derived from dose-response curves of aspirin alone, metformin alone, fluvastatin alone and their combinations (across a concentration range). Values represent average cell survival (%) ± SEM. Half maximal inhibitory concentration (IC50) of MCF10.AT1-R and MCF10.DCIS is significantly different from MCF10.AT1 cells (**p < 0.01 and ***p < 0.001, respectively)

We next examined whether activating AMPK with aspirin was also effective in overcoming adaptive resistance to fluvastatin, a clinically relevant scenario that may have led to the ineffectiveness of fluvastatin in preventing the histological progression of MCF10.AT1-driven xenografts. We first generated a model of adaptive resistance to fluvastatin by continuously exposing MCF10.AT1 cells to increasing doses of fluvastatin (up to 20 μM) to create an MCF10.AT1-R line. MCF10.AT1-R cells showed significantly higher resistance relative to parental MCF10.AT1 cells with IC50 of 8.5 μM compared to 2.1 μM in the parental AT1 line (Fig. 8a). We next tested whether adaptive resistance of MCF10.AT1-R cells to fluvastatin can be overcome with AMPK-activating drugs. Cell proliferation was assayed by performing the MTT assay with a range of aspirin alone, metformin alone and finally fluvastatin with or without simultaneous exposure to aspirin or metformin. Proportions of cell death under each experimental condition were calculated and entered into Calcusyn, software that determines combined drug effects by taking into account the entire shape of the growth inhibition curve and performs multiple drug dose-effect calculations as described by Chou and Talalay [21]. The output of this assay is a “combination index (CI)” that is calculated by the median drug-effect analysis method and suggests whether a drug combination is synergistic (CI < 1), additive (CI = 1) or antagonistic (CI > 1). Through these analyses, we identified a range (that included IC25, IC50, IC75) of aspirin (0.5–10 mM) or metformin (0.5–10 mM) to have CI < 1 and thus be clearly synergistic with fluvastatin (5–100 μM) in MCF10.AT1-R cells (Fig. 8b, and by normalized isobolograms in Additional file 4: Figure S3 A). For generating these combination dose-response curves, the IC50 of aspirin alone, and of metformin alone were also determined from their drug-response curves (Additional file 4: Figure S3 B and C).

**Fig. 9:**
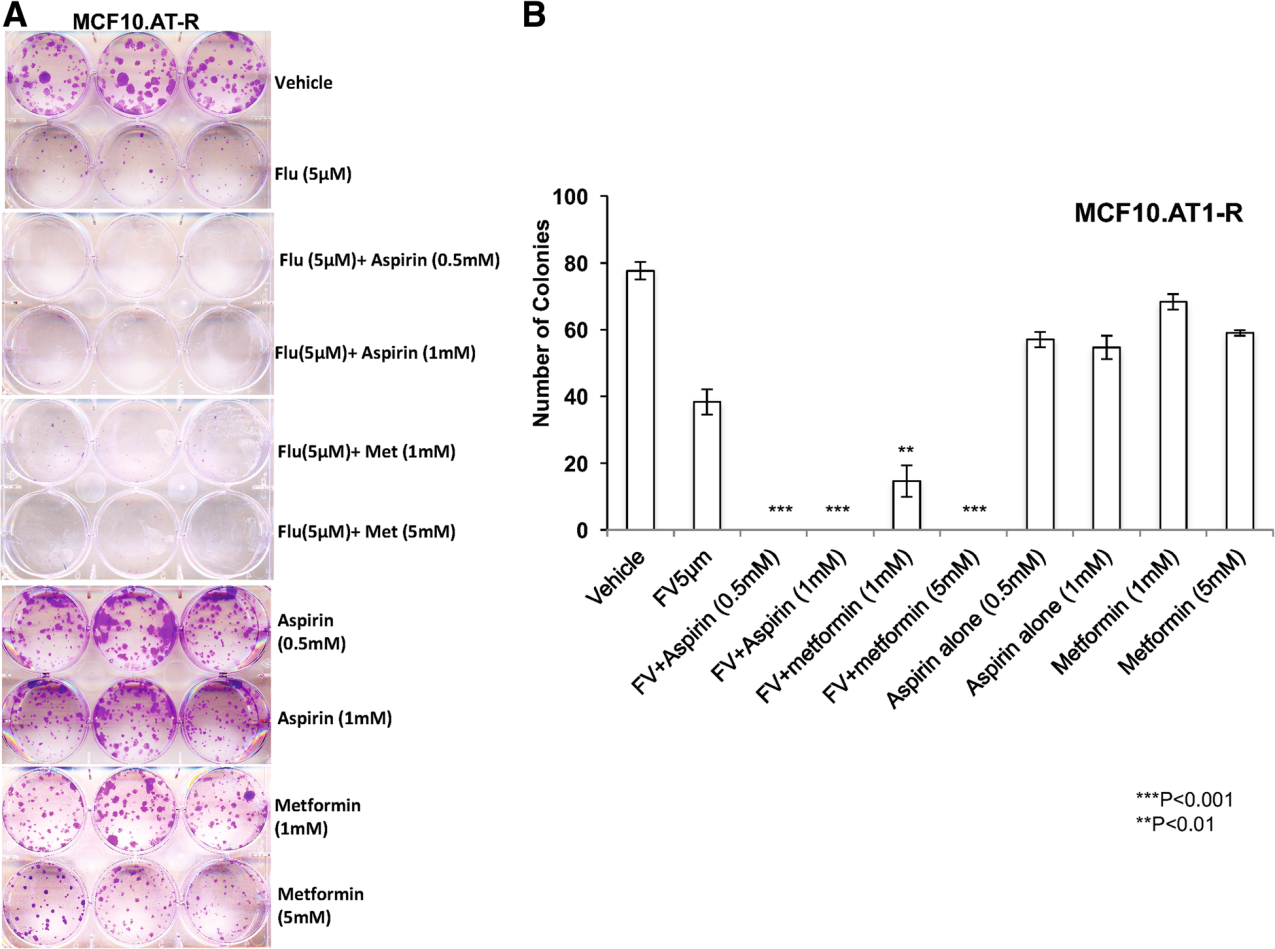
Aspirin and metformin overcome adaptive resistance of MCF10.AT1-R cells. **a**, **b** Images and quantification of the clonogenic survival assay, showing reduction in the number of colonies formed by MCF10.AT1-R cells treated with fluvastatin alone (flu (**a**))or fluvastatin (FV (b)) in combination with aspirin or metformin relative to vehicle control. Colonies ≥2 mm were scored and tabulated. Values represent average number of colonies ± SEM. Drug combinations are compared to fluvastatin alone and are significantly different; **p < 0.01, ***p < 0.001

Last, we tested whether aspirin or metformin in combination with fluvastatin also inhibits the colonizing ability of MCF10.AT1-R (adaptive resistant) cells (Fig. 9). Similar to assays in the inherently resistant MCF10.DCIS line, and consistent with the synergistic interaction between fluvastatin and aspirin/metformin, these assays showed that combination therapy with fluvastatin/aspirin to completely inhibit colony formation (100% inhibition by both 0.5 mM and 1 mM) or fluvastatin/metformin (100% inhibition by 5 mM and 82% inhibition by 1 mM) to be more effective at overcoming adaptive resistance than each drug alone (51% inhibition by fluvastatin, 30% by aspirin and 24% by metformin).

## Discussion

IsomiRNAs have been recently discovered and thus an understanding of their functional relevance in development and cancer is in its infancy. Here we report miR-140-3p-1, an isomiRNA, to be expressed more abundantly than its canonical counterpart (miR-140-3p-2) in a preneoplastic progression model of TNBC, suggesting this isomiRNA is involved in normal breast and TNBC tumorigenesis. While miR-140-3p-1 is expressed relatively more abundantly during the development of TNBC compared to canonical miR-140-3p-2, expression of both miRNAs decreased dramatically from the non-cancer parental line [MCF10A(P)] to the atypia (MCF10.AT1) line, a trend that persisted through subsequent stages to DCIS and invasive cancer (Ca1d). Replacement of miR-140-3p-1 through strand-specific miRNA mimic preferentially inhibited the growth of preneoplastic cells, but not DCIS cells, suggesting its biologic relevance lies primarily in the normal-to-preneoplastic transition and less so in the later stages of disease evolution. Therefore, targeting miR-140-3p-1 and/or its direct gene targets will potentially be most effective in at-risk populations before the onset of DCIS. We found that miR-140-3p-1 controls the mevalonate (MVA) pathway, through direct regulation of *HMGCR* and *HMGCS1*, with the loss of miR-140-3p-1 promoting upregulation of HMGCR and HMGCS1 during the multi-step tumorigenic process. Of interest, and in line with our observations, high expression of MVA pathway genes e.g., *HMGCR*, has been reported to be associated with resistance to therapeutic targeting of the MVA pathway and with poor patient prognosis in breast cancer [18, 22]. Collectively, these observations would suggest that the MVA pathway is involved in breast cancer and a potential target for intervention. Specifically, within the context of preneoplastic disease, this upregulation of the MVA pathway through loss of miR-140-3p-1 creates metabolic vulnerability that may be targeted by repurposing Food and Drug Administration (FDA)-approved low-toxicity drugs such as statins.

Interestingly, the in vitro growth-inhibitory effects of statins were also more prominent in preneoplastic AT1 cells than in DCIS cells, again suggesting that statins are likely to be more effective if given to at-risk patients prior to development of DCIS. However, when we tested whether a statin would inhibit the histological progression of AT1-driven xenografts in mice, our experiments showed that the statin inhibited only the size of the lesions and did not seem to abrogate histological progression towards higher-grade lesions. Statins have been previously shown to inhibit the tumor volume in MCF10NeuA-, MDA-MB-435- and HepG2-driven xenografts [18, 23, 24], which is in agreement with the reduced growth of MCF10.AT1-driven lesions in the current study.

Our data show the complexity of the homeostatic mechanisms that may limit the ability of statins to exploit metabolic vulnerabilities of the transformed cells for purposes of prevention. We found statin treatment alone insufficient to abrogate histologic progression towards cancer, but our data suggest that combination therapy with statin and aspirin or statin and metformin may be a more effective strategy in breast cancer prevention. Our findings also offer a potential explanation of the heterogeneity of the findings noted in epidemiologic studies of statins and breast cancer risk. These population-level data show inconsistent association between statin treatment and breast cancer incidence, with some showing inverse association and others showing no impact of statin use on breast cancer events [22, 25–35]. However, these data do not consider concomitant use of other medications, such as aspirin and metformin. Given the ubiquitous availability of aspirin and given that patients at cardiovascular risk often take both statin and aspirin, it is likely that the available literature on statin use and breast cancer risk is confounded by lack of adjustment for other medications such as aspirin and metformin. Indeed, in one analysis where statin use was found to be associated with reduced risk of breast cancer, the statin-treated group comprised a significantly larger proportion of patients with cardiovascular disease (70% of the statin-user group versus 21% of the control group, *p* < 0.001) and diabetes mellitus (18% of the statin-user group versus 3% of the control group, *p* < 0.001), who are much more likely to also be prescribed aspirin or metformin respectively [31].

Aspirin and metformin are commonly prescribed FDA-approved drugs with acceptable side effects and thus have remained of interest in the domain of breast cancer prevention. Indeed, two current trials are exploring the potential of these agents, administered alone, to reduce breast cancer risk. Alliance A211202 (https://clinicaltrials.gov/ct2/show/NCT01905046?term=A211102&rank=1) is examining the efficacy of metformin (850 mg twice daily) to reverse atypia in the breast, whereas Alliance A211601 (https://clinicaltrials.gov/ct2/show/NCT03609021?term=A211601&rank=1) is exploring the effect of aspirin (300 mg daily), on mammographic breast density, a known breast cancer risk factor. Our data would suggest that although these agents may have a modest effect in inhibiting cell growth, combination therapies that also include statins are likely to have greater efficacy to abrogate growth and presumably greater benefit in reducing cancer risk. In addition to defining optimal combinations of agents for prevention, optimal dosing for these drugs also needs to be explored to determine whether clinically acceptable dosing can modulate the MVA pathway as seen in our preclinical model.

Last, it should also be noted that although our experiments were designed to test the ability of aspirin and metformin to regulate the MVA pathway through AMPK, these drugs (especially at high doses) regulate other pathways in addition to activating AMPK, and in addition AMPK regulates several pathways beyond the MVA pathway; thus, specificity of action and the pleiotropic effects of the aspirin and metformin should be kept in mind while considering their dose and long-term use.

Recently, statin treatments have been suggested to show anticancer effects on tumors that were derived from cells possessing mutated p53 [36–38]. P53 mutants have been shown to aberrantly activate the MVA pathway, and conversely statin inhibition of the MVA pathway is also shown to destabilize and degrade mutated p53, indicating that statins are most likely to work in patients in whom the MVA pathway is activated through p53 mutations. In spite of their wild-type p53 status, MCF10.AT1 cells, the model system used for our xenograft studies, possess an activated cholesterol/ MVA pathway status as indicated by high levels of *HMGCR* and *HMGCS*1, suggesting that other mechanisms must be at play that have activated MVA pathway in our system. We believe that inability of fluvastatin to inhibit the histological progression was due to insufficient suppression of the cholesterol pathway and that activating AMPK will sensitize cells to statins irrespective of p53 status (wild-type or mutant). In agreement with this notion of complete inhibition of the pathway, Penn and coworkers have suggested using dipyridamole, an inhibitor of SREBP cleavage, to prevent SREBP-mediated cholesterol feedback response in addition to statins to increase the therapeutic efficiency of statins alone in mice models [20].

Tumor suppressor gene LKB1, a master protein kinase that governs in the phosphorylation and activation of AMPK, is frequently inactivated in human cancers, including breast cancer. *LKB1* expression negatively correlates with breast cancer stage. Loss of LKB1 disrupts breast epithelial cell polarity and promotes breast cancer cell metastasis and invasion [39]. Therefore, this would suggest that activating AMPK would be appropriate for breast cancer prevention and treatment.

## Conclusions

Our studies suggest that targeting miR-140-3p-1-mediated cholesterol pathway activation by repurposing the FDA-approved, low-toxicity drugs, statins and aspirin, has potential for breast cancer prevention, including TNBC. Interventional trials of a combination of statin and aspirin in women at high risk of breast cancer are needed for further confirmation.

## Additional files

Additional file 1: **Table S1.** qPCR primers. (DOCX 44 kb)

Additional file 2: **Figure S1.** miR-140-3p-1 modestly inhibits proliferation of breast preneoplastic cells. (A) Immunofluorescence-based Ki67 staining of preneoplastic MCF10.AT1 or DCIS cells that were transiently transfected with miR-140-3p-1 mimic or scramble control mimic. (B) Quantification of percentage of inhibition in Ki67-positive (expressing > 3 Ki67 foci) AT1 and DCIS cells with miR-140-3p-1 transfection relative to scramble control mimic transfection. Values represent mean fold change ± SEM. (DOCX 1539 kb)

Additional file 3: **Figure S2.** Cholesterol pathway mediators  *HMGCR* and *HMGCS1* increase during breast cancer progression. (A) Filters to integrate miR-140-3p-1 expression with RNA-seq results of MCF10A breast cancer progression panel to identify functional gene targets of miR-140-3p-1. (B) Top deregulated pathways during breast cancer progression identified using ingenuity pathway analysis. The mevalonate pathway was identified as the top pathway. (C and D) Endogenous *HMGCR* and *HMGCS1* mRNA levels in a MCF10A-based breast cancer progression model. Levels were determined by qPCR. Values are normalized to*RPL19* mRNA levels and represent mean fold change (± SEM) relative to MCF10A(P): *p < 0.05. (PPTX 59 kb)

Additional file 4: **Figure S3.** Aspirin and metformin synergize with fluvastatin to sensitize MCF10.AT1-R and MCF10.DCIS cells. (A) Normalized isobolograms showing a range of fluvastatin and aspirin/metformin to have a combined drug efficacy index (CI) < 1 at multiple doses in MCF10.AT1-R and DCIS cells. Each point within the isobologram represents a treatment combination and its associated number represents a data point for that treatment combination. (B and C) Dose-response curves of aspirin and metformin in MCF10.AT1-R and DCIS cells showing their IC50s that were derived from the MTT assays. (DOCX 354 kb)

## Abbreviations

AMPK: AMP-activated protein kinase
AT1: Atypia
bp: Base pairs
DCIS: Ductal carcinoma in situ
FDA: Food and Drug Administration
HMGCR: HMG-COA reductase
HMGCS1: HMG-COA synthase1
IC50: Half maximal inhibitory concentration
IPA: Ingenuity pathway analysis
LKB1: Liver kinase B1
miRNA: Micro RNA
mRNA: Messenger RNA
MVA: Mevalonic acid
PBS: Phosphate-buffered saline
RPL19: Ribosomal protein L19
SREBP: Sterol regulatory element binding protein 1
TNBC: Triple-negative breast cancer
UTR: Untranslated region
