# Supplementary material for: The isomiR-140-3p-regulated mevalonic acid pathway as a potential target for prevention of triple negative breast cancer
Source: Breast Cancer Res. 2018 Dec 11;20:150. doi: 10.1186/s13058-018-1074-z (PMC6290546; doi:10.1186/s13058-018-1074-z)
Supplement: Supplementary file 1 — Table S1. qPCR primers. (DOCX 44 kb) [file 13058_2018_1074_MOESM1_ESM.docx]

Additional File 4

**Table S1.**
