# Supplementary material for: The isomiR-140-3p-regulated mevalonic acid pathway as a potential target for prevention of triple negative breast cancer
Source: Breast Cancer Res. 2018 Dec 11;20:150. doi: 10.1186/s13058-018-1074-z (PMC6290546; doi:10.1186/s13058-018-1074-z)
Supplement: Supplementary file 2 — Figure S1. miR-140-3p-1 modestly inhibits proliferation of breast preneoplastic cells. (A) Immunofluorescence-based Ki67 staining of preneoplastic MCF10.AT1 or DCIS cells that were transiently transfected with miR-140-3p-1 mimic or scramble control mimic. (B) Quantification of percentage of inhibition in Ki67-positive (expressing > 3 Ki67 foci) AT1 and DCIS cells with miR-140-3p-1 transfection relative to scramble control mimic transfection. Values represent mean fold change ± SEM. (DOCX 1539 kb) [file 13058_2018_1074_MOESM2_ESM.docx]

Additional File 1

**
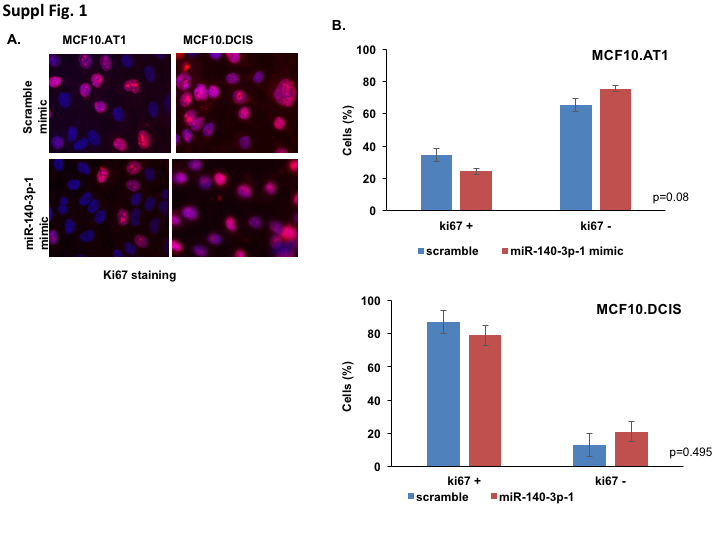
Figure S1.** **miR-140-3p-1 modestly inhibits proliferation of breast preneoplastic cells.** **A,** Immunofluorescence-based Ki67 staining of preneoplastic MCF10.AT1 or DCIS cells that were transiently transfected with miR-140-3p-1 mimic or scramble control mimic. **B,** Quantification of percentage of inhibition in Ki67-positive (expressing >3 Ki67 foci) AT1 and DCIS cells with miR-140-3p-1 transfection relative to scramble control mimic transfection. Values represent mean fold change ± SEM.
