# Supplementary material for: The isomiR-140-3p-regulated mevalonic acid pathway as a potential target for prevention of triple negative breast cancer
Source: Breast Cancer Res. 2018 Dec 11;20:150. doi: 10.1186/s13058-018-1074-z (PMC6290546; doi:10.1186/s13058-018-1074-z)
Supplement: Supplementary file 3 — Figure S2. Cholesterol pathway mediators HMGCR and HMGCS1 increase during breast cancer progression. (A) Filters to integrate miR-140-3p-1 expression with RNA-seq results of MCF10A breast cancer progression panel to identify functional gene targets of miR-140-3p-1. (B) Top deregulated pathways during breast cancer progression identified using ingenuity pathway analysis. The mevalonate pathway was identified as the top pathway. (C and D) Endogenous HMGCR and HMGCS1 mRNA levels in a MCF10A-based breast cancer progression model. Levels were determined by qPCR. Values are normalized toRPL19 mRNA levels and represent mean fold change (± SEM) relative to MCF10A(P): *p < 0.05. (PPTX 59 kb) [file 13058_2018_1074_MOESM3_ESM.pptx]

## Slide 1
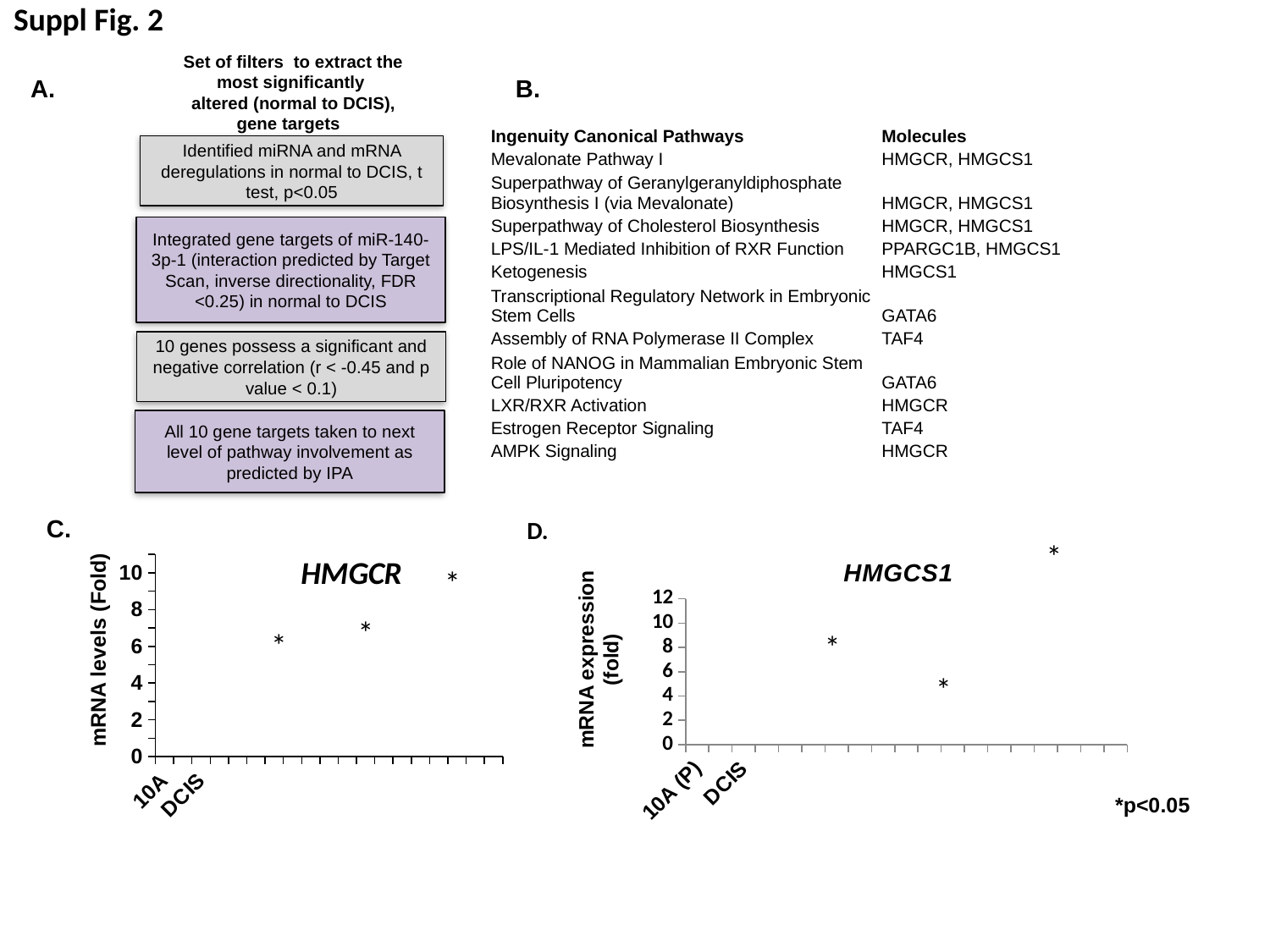

Suppl Fig. 2
Set of filters to extract the most significantly
altered (normal to DCIS), gene targets
A.
B.
| Ingenuity Canonical Pathways | Molecules |
| --- | --- |
| Mevalonate Pathway I | HMGCR, HMGCS1 |
| Superpathway of Geranylgeranyldiphosphate Biosynthesis I (via Mevalonate) | HMGCR, HMGCS1 |
| Superpathway of Cholesterol Biosynthesis | HMGCR, HMGCS1 |
| LPS/IL-1 Mediated Inhibition of RXR Function | PPARGC1B, HMGCS1 |
| Ketogenesis | HMGCS1 |
| Transcriptional Regulatory Network in Embryonic Stem Cells | GATA6 |
| Assembly of RNA Polymerase II Complex | TAF4 |
| Role of NANOG in Mammalian Embryonic Stem Cell Pluripotency | GATA6 |
| LXR/RXR Activation | HMGCR |
| Estrogen Receptor Signaling | TAF4 |
| AMPK Signaling | HMGCR |
Identified miRNA and mRNA deregulations in normal to DCIS, t test, p<0.05
Integrated gene targets of miR-140-3p-1 (interaction predicted by Target Scan, inverse directionality, FDR <0.25) in normal to DCIS
10 genes possess a significant and negative correlation (r < -0.45 and p value < 0.1)
All 10 gene targets taken to next level of pathway involvement as predicted by IPA
C.
D.
### Chart: HMGCR
| Category | |
|---|---|
| 10A (P) | 1.0 |
| AT1 | 1.5 |
| DCIS | 1.7 |
| Ca1d | 2.46 |
### Chart: HMGCS1
| Category | |
|---|---|
| 10A (P) | 1.0 |
| AT1 | 3.18 |
| DCIS | 2.1 |
| Ca1d | 5.5 |*
*
*
*
*
mRNA expression (fold)
mRNA levels (Fold)
*
*p<0.05
