# Supplementary material for: The isomiR-140-3p-regulated mevalonic acid pathway as a potential target for prevention of triple negative breast cancer
Source: Breast Cancer Res. 2018 Dec 11;20:150. doi: 10.1186/s13058-018-1074-z (PMC6290546; doi:10.1186/s13058-018-1074-z)
Supplement: Supplementary file 4 — Figure S3. Aspirin and metformin synergize with fluvastatin to sensitize MCF10.AT1-R and MCF10.DCIS cells. (A) Normalized isobolograms showing a range of fluvastatin and aspirin/metformin to have a combined drug efficacy index (CI) < 1 at multiple doses in MCF10.AT1-R and DCIS cells. Each point within the isobologram represents a treatment combination and its associated number represents a data point for that treatment combination. (B and C) Dose-response curves of aspirin and metformin in MCF10.AT1-R and DCIS cells showing their IC50s that were derived from the MTT assays. (DOCX 354 kb) [file 13058_2018_1074_MOESM4_ESM.docx]

Additional File 3

**Figure S3.** **Aspirin and metformin synergize with fluvastatin to sensitize MCF10.AT1-R and MCF10.DCIS cells.** **A,** Normalized isobolograms showing a range of fluvastatin and aspirin / metformin to have a combined drug efficacy index (CI) to be less than 1 at multiple doses in MCF10.AT1-R and DCIS cells. Each point within the isobologram represents a treatment combination and its associated number represents a data point for that treatment combination **B&C,** Dose response curves of aspirin and metformin in MCF10.AT1-R and DCIS cells showing their IC50s that were derived from their MTT assay.
